# Neonatal Colostrum Intake in Dairy Heifers Can Affect Lactation Performance

**DOI:** 10.3390/ani16213461

**Published:** 2026-11-02

**Authors:** Rebel Skirving, Cynthia D. K. Bottema, Do Thi Hue, Richard Laven, Kiro R. Petrovski

**Affiliations:** 1 Davies Livestock Research Centre, School of Animal & Veterinary Sciences, Adelaide University, Roseworthy Campus, Mudla Wirra Rd, Roseworthy 5371, Australia; rebel.skirving@adelaide.edu.au (R.S.); cynthia.bottema@adelaide.edu.au (C.D.K.B.); hue.do@adelaide.edu.au (D.T.H.); 2 Gambier Vets Pty. Ltd., 22 Allison Street, Mount Gambier 5290, Australia; 3 School of Veterinary Science, Massey University, Private Bag 11-222, Palmerston North 4442, New Zealand; r.laven@massey.ac.nz; 4 Australian Centre for Antimicrobial Resistance Ecology, School of Animal & Veterinary Sciences, Adelaide University, Roseworthy Campus, Mudla Wirra Rd, Roseworthy 5371, Australia

**Keywords:** immunoglobulin, epigenetics, milk production, total milk protein, bovine

## Abstract

Passive transfer of immunity through colostrum is known to improve short-term health in neonatal dairy calves, but its long-term effects remain poorly understood. Lactational programming proposes that bioactive components in milk and colostrum induce epigenetic changes that influence future performance, although this concept has received limited attention in dairy cattle. The aim of this study was to investigate potential associations between neonatal colostrum intake, as estimated by serum total protein (STP), with subsequent growth, reproductive performance, and milk production in 2661 dairy heifers from five pasture-based dairy herds in South Australia. Blood samples collected within the first 1–7 days of life were used to measure STP as an indicator of passive immunity transfer, and consequently, colostrum intake. Heifers with adequate passive transfer (STP > 50 g/L) produced 0.5, 2.4, and 0.7 kg more milk per day during their first, second, and third lactations, respectively, than heifers with inadequate passive transfer (STP ≤ 50 g/L). In contrast, no meaningful associations were identified between neonatal STP and yearling weight, age at first or subsequent calvings or calving intervals. These findings suggest that adequate colostrum intake enhances lifetime milk production and provide evidence supporting lactocrine programming in dairy cattle.

## Simple Summary

When calves are born, they do not have immunity to specific infectious diseases. To obtain this immunity, they must consume their dam’s first milk, known as colostrum. This is because colostrum not only contains nutrients but also immune components and growth factors. Hence, good colostrum intake helps prevent infectious disease in the calves in addition to helping their growth and development. In other species such as pigs, colostrum has been shown to have longer term effects as well (for example, increased fertility as adults). The study herein investigated whether colostrum consumption also has longterm effects in cattle. The most significant finding from the study was that when female dairy calves receive enough good quality colostrum as newborns, then they will produce more milk as dairy cows.

## 1. Introduction

Neonatal calves are born agammaglobulinemic [1,2], and are at a high risk of infectious diseases unless they can absorb essential immunoglobulins and other immune components from colostrum within their first 24–36 h of life [3,4]. The intake of adequate amounts of good quality colostrum, within the right time-frame, has been shown to be beneficial to calf health and growth in the first few weeks of life [5]. Raising female dairy calves is expensive, time-consuming and environmentally challenging, but critical to the success of dairy enterprises, as these calves ultimately become the new milking herd [6,7,8,9]. Modern dairy operators, therefore, aim to raise heifers efficiently and humanely, with an emphasis on increased average daily weight gain (growth rates), high rates of fertility, maximal lactation results, decreased time to first calving, and improved longevity [6]. To achieve these results, calves must be as healthy as possible, and a critical moment in their neonatal health is the absorption of immunoglobulins and other immune components from colostrum.

The immune benefits of colostrum to neonatal calves have been well recognized for decades, and more recently, it has been demonstrated that colostrum contains other bioactive substances, such as growth hormones, proteins and microRNA (miRNA) [10,11,12,13]. Such substances can be absorbed into the calf’s circulation in their active form where they may exert an immediate effect on the calf [14]. The concept of environmental influences being able to alter phenotype, sometimes referred to as “developmental plasticity” [9], is at the heart of the study of epigenetics. In mammals, a neonatal milk diet has the potential to modify phenotypes, and this concept has been referred to as the “lactocrine hypothesis” [15] or “lactocrine programming” [16]. Porcine studies have shown the neonatal intake of colostrum can affect development of the reproductive tract in male and female piglets [9,16,17]. However, while lactocrine programming is plausible in cattle, it remains an unproven hypothesis [18]. The expense and complexity of longitudinal studies in bovine species have led to only a limited understanding of the potential long-term effects of the transfer of passive immunity and other bioactive substances from colostrum on the development and productivity of cattle.

In the few studies to explore the long-term effects of colostrum intake and transfer of passive immunity on cattle, the results are equivocal. The studies that compared passive immunity status in neonates with reproductive parameters found serum immunoglobulin (Ig) levels did not have a clear effect on the age at first calving [19] or on the odds of being diagnosed pregnant (OR = 1.44; 95% CI [0.82, 2.69]) [20]. Another study that compared calves fed 4 L colostrum with calves fed 2 L colostrum found no important difference in the age at conception, although it should be noted serum protein/Ig levels were not measured in this study [21]. Interestingly, a more recent study demonstrated that when heifers with poor transfer of passive immunity as neonates were compared to those with excellent transfer of passive immunity, they were less likely to remain in the herd until the time of first insemination, successful insemination or first calving [22].

In the longitudinal studies that compared colostrum intake and passive immunity status with lactation performance, the results were interesting and variable. DeNise et al. (1989) demonstrated that for each increase in immunoglobulin unit, heifers had an increase of 8.5 kg “mature-equivalent milk” and 0.24 kg “mature-equivalent fat” per day during their first lactation [19]. A small case study that compared heifers fed 4 L colostrum to heifers fed 2 L colostrum found the former group produced more milk than the latter group in the first two lactations (9907 ± 335 and 11,294 ± 335 kg vs. 8952 ± 341 and 9642 ± 341 kg, respectively). A limitation of this second study was the lack of an indication of the colostrum intake (e.g., serum protein/Ig levels) [21]. The group fed 4 L colostrum also had greater average daily weight gain compared to the group fed 2 L colostrum (1.03 ± 0.03 kg vs. 0.80 ± 0.02 kg), which may have influenced milk production. Another study that examined the effect of early-life events on lactation performance acknowledged transfer of passive immunity as a predictor of lactation performance; however, the passive immunity status of calves was not included in the final model [23]. A longitudinal study of dairy cattle in New Zealand found no important association between passive immunity status and milk solids production (5 g; 95% CI [−1.6, 27]) [20]. Similarly, a single-herd study on dairy cattle in America demonstrated no appreciable difference between heifers from different transfer of passive immunity groups and their first-lactation 305-day mature-equivalent milk production [22].

These studies highlight the lack of data and clarity surrounding the potential long-term effects of colostrum intake and/or transfer of passive immunity on dairy cattle reproductive and lactation performance. Therefore, the aims of this study were to explore the potential relationship between neonatal colostrum intake (as estimated by serum total protein (STP) concentration) and bodyweights at approximately one year of age, age at each calving, the interval between calvings, and lactation performance in heifers from five pasture-based herds in the southeast of South Australia.

## 2. Materials and Methods

All animal handling and testing procedures were approved by the Animal Ethics Committee of Adelaide University (S-2017-060, S-2021-011 and S-2021-049). The owners of all animals enrolled in this study gave informed consent regarding the use of their cattle for research.

This longitudinal, prospective, observational study was conducted on five pasture-based dairy farms from the Mount Gambier region of southeast South Australia. The climate is Mediterranean and there was an average annual rainfall of 715 mm over the period of this study, from February 2018 to September 2025 [24]. The breeds of cattle in this study were Holstein-Friesian, Jersey and their crosses. Calves were individually identified by ear-tag numbers and/or names and National Livestock Identification System (NLIS) electronic ear-tags.

This study was undertaken on farms within a 50 km radius of Mount Gambier, South Australia. Farms were selected for calf rearing and colostrum management protocols that were representative of those commonly used in the district. Calves were born during one season (July to October) on Farms 2 and 3, while on Farms 1, 4 and 5, calves were born during two seasons (July to December or January to May). Calves from the farms with two calving seasons were grouped as “cohorts”, according to their season of birth (Supplementary Table S1). Therefore, there were 8 separate cohorts of calves. Calves on two farms were collected from their dams within 24 h post-partum (Farms 2 and 3), and on the other three farms (Farms 1, 4 and 5) within 12 h post-partum. Calves were fed immediately after separation from their dams. For their first feed after separation from their dam, calves from three farms (Farms 2, 4 and 5) were given colostrum only, which was collected from cows within 12 h post-partum, whereas on the other two farms (Farms 1 and 3), the calves were given a mixture of colostrum and transition milk (collected from cows 12 h to 4 days post-partum). If the colostrum was not fed fresh, it was either refrigerated or frozen but was not routinely pasteurized. Farm 4 was the only one where colostrum quality was measured before feeding calves, and on this farm, only good quality colostrum was used (>22% Brix) (Supplementary Table S1). All calves were reared away from their dams, in dedicated open-sided calf sheds. After weaning, all heifers were reared within 100 km of the farm of origin, and all had access to pasture all year round, each year.

### 2.1. Sample Collection and Measurements

Eligible female calves born on these farms over a period from February 2018 to May 2021 were blood tested at 1–7 days of age (n = 2661) and had serum total protein (STP) measured by digital refractometry, as an estimate of the transfer of passive immunity and potential uptake of compounds/substances from the ingested colostrum [25,26,27]. To validate the use of STP as an estimate of passive immunity transfer herein, serum samples from a subset of the calves with a range of STP levels were measured for IgG concentrations [27]. Calves older than 7 days were excluded from the study to ensure that the STP levels accurately reflected the transfer of IgG as an estimate of colostrum intake [28,29]. 

An experienced dairy veterinarian assessed the health and hydration of each of the enlisted calves. Any calves deemed to be sick or dehydrated, based on physical examination and rectal temperature, were excluded from this study. Heifers ranged from 280–518 days (9–16 months) when weighed as yearlings by the farm managers. All five farms had records for yearling weights (n = 1884) and calving data (n = 1389). Culled animals were defined in this study as those not present at set time-points, and included mortalities, sales, and removals based on herd selection criteria (e.g., conformation, temperament). Lactation results were available for two farms from a Lely automatic milking system (Lely Industries N.V., Netherlands), which recorded every milking (Farm 4, n = 89,584 records) or from periodic herd testing (Farm 5, n = 1164 records).

#### 2.1.1. Weight Measurement at Approximately One Year of Age

Each calf cohort was mustered once only at approximately one year of age (Supplementary Table S1). Mustering was timed to coincide with husbandry procedures routinely performed on each farm, such as vaccination, parasite treatment and/or selection for mating. Calves were individually identified and weighed on portable cattle scales (Tru-Test Ez-Weigh scales, Datamars, Australia).

#### 2.1.2. Data Collection for Calving Events

The date of first calving for each heifer was recorded on the respective farms by the herd managers, at the time of calving. These data were matched to each heifer’s records, and the age (in days) at first calving was calculated as the difference in days between the heifers’ date of birth and the recorded date of her first calving. For subsequent calvings, calving dates were again recorded by the herd managers and the interval between calvings calculated as the difference in days between one calving and the subsequent calving (i.e., first calving date to second calving date and second calving date to third calving date).

### 2.2. Statistical Analysis

Calves were classified by their colostrum intake according to neonatal serum total protein (STP) concentrations using the dichotomous standard of adequate colostrum intake (STP > 50 g/L) or poor colostrum intake (STP ≤ 50 g/L) [30]. The “adequate” category includes calves that received “fair”, “good” and “excellent” transfer of passive immunity from the intake of colostrum, as reviewed by Lombard et al. (2020) [30].

The effect of STP category on weight at approximately 12 months old (~12 months old) was analyzed using a generalized estimating equation (GEE) in SPSS (version 30, IBM, New York, NY, USA) with cohort within farm as the subject variable and heifer identification name or number (ID) as the within-subject variable. Weight was the linear outcome variable, and cohort and STP category (adequate vs. poor) were the predictor variables. For this and all subsequent GEE, an independent working correlation matrix, was used alongside a robust estimator for the covariance matrix.

As age at culling was not recorded, the association between STP category and age at first calving was analyzed using three models. The first model focused on whether any heifer with an STP result was recorded as calving for the first time on the farm of origin. This binomial outcome (recorded vs. not recorded) was analyzed using a GEE with cohort within farm as the subject variable and heifer ID as the within-subject variable. The predictor variables were cohort and STP category (adequate vs. poor). The second model focused on whether a calf was present at the ~12-month weighing. The outcome was again binomial (recorded vs. not recorded) and analyzed using a GEE with cohort within farm as the subject variable and calf ID as the within-subject variable. The predictor variables for this second model were cohort, ~12-month weight, and STP category (adequate vs. poor). The final model was a Cox proportional hazards survival model with age at first calving as the outcome variable and cohort within farm, ~12-month weight and STP category as the predictor variables. For the second and third models, heifers who had an age at first calving but no weight data at ~12 months were excluded from the analysis. For this and subsequent Cox proportional hazards survival models, the proportional hazards assumption was assessed visually using Kaplan–Meier curves and log(−log) plots. For the subsequent calving intervals, two Cox proportional hazards survival models were used. The first “full” model had calving interval (days between recorded calvings) as the outcome variable and cohort within farm, ~12-month weight, interval between calving and start of the breeding season and STP category as the predictor variables. The second model had STP category as the only predictor variable. For all Cox survival models, data were interpreted using the point estimate of the HR and its 95% confidence intervals (CI). HR whose 95% CI were completely between 0.85 and 1.2 were considered to indicate associations that were small, and thus of limited importance, whereas HR whose 95% CI included values between 0.5 and 0.84 or between 1.3 and 2.0 were considered to indicate associations that were moderate, and thus potentially important.

Fat- and protein-corrected milk (FPCM) yields (FPCM (kg) = Milk (kg) × [0.1226 × Fat (%) + 0.0776 × True Protein (%) + 0.2534]) were used to create lactation curves [31,32] using the modified Ali–Shaeffer model [33]. Milk production was standardized to a 305-day lactation. Outputs from the Ali–Shaeffer curves were used in a separate mixed model in the evaluation of the association between serum total protein (STP) categories and milk production per farm. The preliminary model included breed as a fixed effect. However, breed was not significant after the fat and protein correction, and therefore, breed was removed from the final model. The outputs were the fat- and protein-corrected milk production means and their standard errors. The outputs were used to run a separate linear mixed model in PROC MIXED to evaluate the association between serum total protein (STP) category and milk production per lactation within a farm. The outputs were the daily fat- and-protein-converted milk production means and their standard errors.

## 3. Results

In order to determine colostrum intake in the calves, serum total protein (STP) levels were determined as a surrogate for IgG serum concentration and assigned as poor or adequate colostrum based on the thresholds from Lombard et al. (2020) [30]. Of the 2661 female calves enrolled in this study, there were 1443 with STP > 50 g/L (indicating adequate transfer of passive immunity from colostrum intake) and 1218 with STP ≤ 50 g/L (indicating poor transfer of passive immunity) (Table 1). Of all the heifers in this study, 74.4% were from Farm 1, a large commercial dairy, whereas the remaining 25.6% were from four smaller, family-owned dairy farms.

### 3.1. Descriptive Statistics

At the time of neonatal sampling, the percentage of heifer calves with poor transfer of passive immunity (STP ≤ 50 g/L) by farm ranged from 6% on Farm 4 to 53% on Farm 3 (Table 1). The proportion of heifers that remained on the farms and had a recorded weight at ~12 months ranged from 38% on Farm 2 to 98% on Farm 4, while the proportion of heifers which had a recorded first calving date ranged from 32% on Farm 2 to 96% on Farm 4 (Table 1).

### 3.2. Effect of STP Category on Weight at ~12 Months of Age

**Figure 1:**
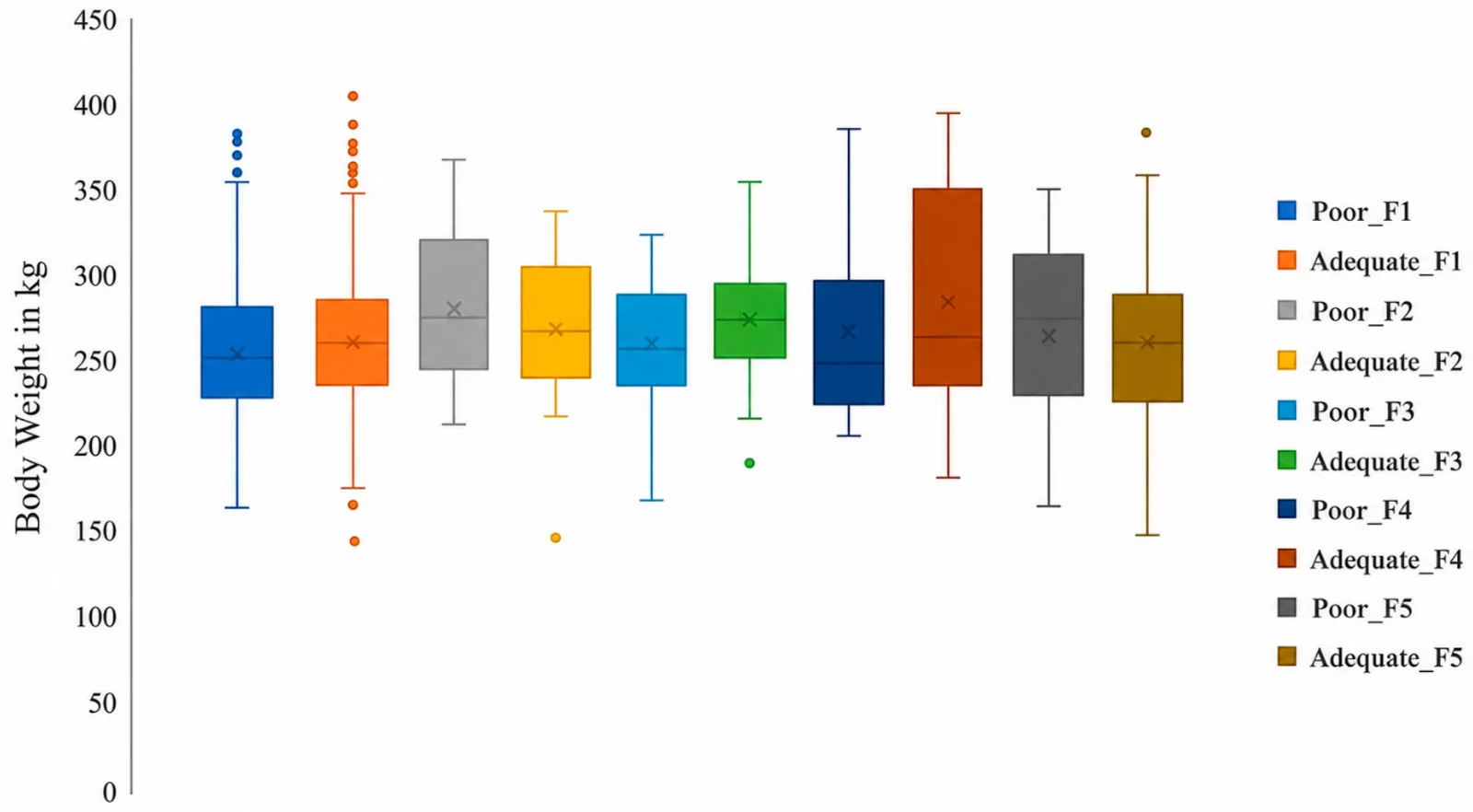
Raw data showing the weight distribution of heifers at approximately one year of age. Box plots represent the “poor” and “adequate” colostrum intake groups from each of the five farms (F1–F5).

Heifer weight at ~12 months of age varied within and between farms as well as within cohorts within farms (Figure 1, Supplementary Table S2). After accounting for cohort, heifers with poor colostrum intake (neonatal STP ≤ 50 g/L) were 4.2 kg (95% CI [0.35, 8.63]) lighter than heifers with adequate colostrum intake (STP > 50 g/L). The estimated marginal means from the model were 279.7 kg (95% CI [278.3, 281.1]) for heifers with neonatal STP > 50 g/L and 275.5 kg (95% CI [272.4, 278.6]) for heifers with STP ≤ 50 g/L. Overall, the data for heifers with poor colostrum intake were consistent with a range of effects from a small and economically unimportant increase in weight to a moderate decrease.

### 3.3. Reproductive Parameters

#### 3.3.1. Effect of STP Category on Age at First Calving

Across all farms, recorded age at first calving ranged from 631 to 1443 days (Supplementary Table S3). At the individual farm level, median recorded age at first calving ranged from 721 days (Farm 2) to 803 days (Farm 5) (Supplementary Table S3).

Across all heifers, after accounting for cohort, having a neonatal STP ≤ 50 g/L reduced the odds of having a first calving record compared to heifers with neonatal STP > 50 g/L (OR = 0.76, 95% CI [0.64, 0.91]). After accounting for cohort, the model estimated that the mean probability of having a recorded first calving was 63% (95% CI [60, 65]) if neonatal STP was ≤50 g/L compared to 69% (95% CI [67, 70]) if STP > 50 g/L.

Across all heifers with a weight record at ~12 months of age, after accounting for cohort and weight at ~12 months, the model did not identify an important effect of neonatal STP category on the odds of a heifer being recorded as having a first calving (OR = 1.0, 95% CI [0.86, 1.15]) if the neonatal STP was ≤50 g/L vs. >50 g/L. The mean probability of having a recorded calving for a heifer with mean weight of 265 kg was 78% (95% CI [76, 80]) if the neonatal STP was ≤50 g/L compared to 78% (95% CI [77, 80]) if the neonatal STP was >50 g/L. In contrast, an increase of 10 kg in bodyweight at ~12 months was associated with increased odds of having a recorded first calving (OR = 1.1, 95% CI [1.02, 1.2]), although the identified effect was small.

With regard to age at first calving, after accounting for cohort and weight at ~12 months, the survival model indicated that heifers whose neonatal STP was ≤50 g/L had a later calving date than heifers whose STP > 50 g/L. However, the differences were small, and not consistent with an important impact of poor colostrum intake on age at first calving (HR = 0.95, 95% CI [0.85, 1.07]). Increased weight at ~12 months of age was associated with an increase in age at first calving (HR = 0.96 for an increase in weight of 10 kg, 95% CI [0.94, 0.98]), though the effect was small.

#### 3.3.2. Effect of STP Category on Reproductive Performance After the First Calving

Of the 1389 heifers across all five farms with a first calving date, 1121 (81%) had a second recorded calving date. Data from Farm 3 were not available for the cows beyond the second calving date. However, for the remaining four farms with third calving dates, there was a total of 666 cows with records (48%). Only Farm 1 provided data/dates for cows with a fourth and a fifth calving, n = 256 (18%) and n = 65 (5%), respectively. As mating on Farm 5 was not truly seasonal, data from this farm were excluded from this analysis.

##### 3.3.2.1. First Calving Interval

Across Farms 1 to 4, 1019 cows had a recorded second calving date with the interval between first and second calving (first calving interval) ranging from 304 days to 1291 days. Median calving interval by farm ranged from 370 days (Farm 2) to 386 days (Farm 3).

Of these 1019 cattle with a first calving interval, 979 had weight data (~12 months of age) and calved for the first time before the start of the next breeding season, and therefore, could be included in the analysis. In the full model, after accounting for cohort, weight at ~12 months had no important impact on calving interval as the hazard ratio for an increase of 10 kg in bodyweight was 1.01 (95% CI [0.99, 1.04]), while an increased interval between calving and start of the breeding season was associated with a small increase in first calving interval (HR = 0.92 for an increase of 20 days, 95% CI [0.86, 0.98]). In this model, the neonatal STP had no important impact on calving interval as the HR for heifers with STP ≤ 50 g/L compared to heifers with STP > 50 g/L was 0.98 (95% CI [0.86, 1.12]). Using the same model with all 1019 cattle with first calving interval data, where the predictors variables were only cohort and STP category, did not change this conclusion between heifers with STP ≤ 50 g/L compared to heifers with STP > 50 g/L (HR = 0.97, 95% CI [0.85, 1.11]).

##### 3.3.2.2. Second Calving Interval

Of the 639 cows recorded on Farms 1, 2 and 4 as having a third calving date, 581 came from Farm 1, 33 from Farm 2 and 25 from Farm 4. Therefore, the second calving interval (that is, the interval between second and third calving) was evaluated using data only from Farm 1. The median second calving interval was 372 days (range = 301–753). Of the 581 cattle on Farm 1 with a second calving interval, 464 calved for the second time prior to the start of the subsequent breeding season and could be included in the analysis. In the full model, after accounting for cohort, neither weight at ~12 months nor the interval between calving and the start of the breeding season had an important impact on second calving interval as the HR for an increase of 10 kg in bodyweight was 0.98 (95% CI [0.95, 1.01]), and the HR for an increase of 20 days to the start of breeding season was 0.97 (95% CI [0.92,1.10]. In this model, the effect of neonatal STP ≤ 50 g/L did not have a clear impact on calving interval (HR = 1.1, 95% CI [0.92, 1.33]), with the effect ranging from a small decrease in HR to a moderate increase. The survival model with STP category as the only predictor of second calving interval did not change this conclusion (HR = 1.04, 95% CI [0.88, 1.23]).

### 3.4. Milk Production Parameters

From the comprehensive milk production data available from two farms (Farm 4 and 5), the average daily milk production over the three lactations differed between farms, lactations, and serum total protein (STP) category (Table 2). The total average daily milk production over the three lactations was 24.7 and 27.9 kg of fat-and-protein-converted milk for Farms 4 and 5, respectively, which differed significantly between the farms (*p* < 0.001). The total average daily milk production over the three lactations was 24.4 and 25.6 kg of milk for cows in the poor and adequate colostrum intake categories (*p* < 0.001). On average, cows from the poor category (STP ≤ 50 g/L) produced 0.5, 2.4, and 0.7 kg less milk per day in the first, second and third lactation, respectively, compared to those in the adequate colostrum intake category (STP > 50 g/L). Cows in each STP category consistently increased their milk daily average production at each subsequent lactation.

## 4. Discussion

The aim of this study was to investigate the potential relationship between neonatal colostrum intake (as estimated by serum total protein (STP) concentration) and heifer weights at approximately one year of age, heifer/cow age at each calving, the interval between calvings, and lactation performance across three lactations. It was conducted on female cattle from five pasture-based dairy herds in the southeast region of South Australia, where, as neonatal calves, 46% of them had poor colostrum intake (neonatal serum total protein (STP) ≤ 50 g/L) and 54% had adequate colostrum intake (STP > 50 g/L). The calves with poor colostrum intake appeared to perform comparably with calves that had adequate colostrum intake with regard to yearling weights, age at each calving and calving-to-calving intervals. However, there was a notable difference between heifers in the two colostrum intake categories with regard to lactation performance. Cows that had adequate colostrum intake as neonates had better lactation performance in their first three lactations compared to cows with poor neonatal colostrum intake. The precise mechanisms behind these observed effects remain unclear. They may stem directly from poor transfer of passive immunity or indirectly through the impact of poor transfer of passive immunity on heifer growth and health. The differences may also be the result of insufficient uptake of other bioactive compounds present in colostrum (i.e., the lack of lactocrine programming) or a combination of these factors. Given the observational nature of this study, the primary aim was not to establish definitive cause-and-effect relationships for the parameters examined, but to determine if any relationships exist. Further study is now needed to identify possible causes for the observations of this study.

### 4.1. Heifer Weights at Approximately One Year of Age

After accounting for cohort, heifer weights at approximately one year of age showed heifers with poor intake of colostrum as neonates were marginally lighter than those with adequate intake of colostrum. This observation is in agreement with other studies that have found adequate colostrum intake (as measured by the transfer of passive immunity and assumed to be proportional to other bioactive substances) to be beneficial for pre- and post-weaning weights [34,35].

Although the results indicate heifers with poor neonatal colostrum intake were lighter than heifers with adequate neonatal colostrum intake, the effect was small and not likely to be of biological significance. This is consistent with other published studies that also noted no meaningful difference between growth rates and pre- or post-weaning weights in groups of calves that had poor versus adequate colostrum intake [20,22,36]. This incongruity in the literature suggests that other factors related to heifer management on individual farms may have larger effects on yearling weights than colostrum intake alone and warrant further investigation.

### 4.2. Loss of Animals from the Farm of Origin

The average rate of loss from the farm of origin was much higher than expected. It has been reported that between 10–33% of replacement heifers are culled before their second lactation [6,9]. However, in this study, of the original heifer calves sampled as neonates, only 52% were recorded as having a first calving date. The percentage of animals still on the farm of origin with a first calving date recorded varied considerably across the enlisted properties though, from 32% on Farm 2 to 96% on Farm 4. One of the key reasons for the high loss rate herein may have been the unusually high market prices for dairy heifers sold for export. Managers on some of the farms took the opportunity to sell heifers to the export market, thereby dramatically reducing their replacement cow population. It is likely that heifer export sales drove much of the large difference in loss rates between the farms (Table 1). Other reasons for heifers failing to have a first calving date recorded included illness, injury, death or elective culling (e.g., based on temperament, conformation or breeding selection).

Heifers with poor neonatal intake of colostrum were less likely to have a first calving date recorded, when compared to those with adequate neonatal intake of colostrum (63% vs. 69% respectively). However, if the heifers were still in the herd of origin at approximately one year of age, the neonatal STP category did not appear to affect their chance of having a first calving recorded. This implies that STP category had a more pronounced effect on the heifers in the first 12 months of life, but if the heifers remained long enough to record a yearling weight, the STP category did not appear to have an important effect on the likelihood of calving. This observation is in agreement with other published studies that have noted increased morbidity and mortality rates in dairy heifers with inadequate transfer of passive immunity in the first 6 months of life [22,37].

### 4.3. Reproductive Parameters

The reproductive parameters measured in this study were the heifer’s age at first calving and the first and second calving intervals. The heifer’s neonatal STP category had no clear effect on the age at first calving. Similarly, STP category did not have a meaningful effect on the first or second calving intervals. Again, these results suggest that if a heifer is still present in the herd of origin at approximately one year of age, the level of neonatal passive transfer had no meaningful effect on her reproductive performance. The results are in agreement with previous studies that found no significant effect of neonatal transfer of passive immunity on reproductive performance using a range of measures such as age at conception [21], age at first calving [19,21] and number of inseminations [22]. Similar studies conducted in seasonal calving systems also demonstrated a lack of relationship between transfer of passive immunity from colostrum intake and reproductive measures such as the odds of being diagnosed pregnant [20].

Taken together, these data suggest that a long-term effect of poor colostrum intake on reproductive performance is unlikely, especially in pasture-based dairy heifers in South Australia. However, it would be of interest in future studies to examine potential relationships between colostrum intake categories and other areas of reproduction, such as abortions and the effectiveness of estrus synchronization programs.

### 4.4. Milk Production

In contrast to reproductive performance, increased intake of colostrum was associated with increased milk production. More specifically, heifers/cows with adequate intake of colostrum in the current study showed approximate increases of 152, 732 and 214 kg in fat and protein-corrected milk yields per cow in the first, second, and third lactations, respectively. This study was the first to demonstrate this effect across three successive lactations. Other published studies noted a positive relationship between neonatal colostrum intake/transfer of passive immunity and lactation performance across a single lactation [19,38] or across two lactations [21]. However, limitations in these previous studies include low numbers of cows (n = 68) [21], single-herd studies [19,21], no quantification of serum total protein or IgG concentration [21], or not including STP concentration/category in the final model [23]. This was also the first study of its kind to be conducted on pasture-fed dairy cattle from South Australia.

The observed differences in milk production between heifers from different colostrum intake categories in both the current and previous studies raise the possibility of early-life programming through colostrum intake as an influence on future lactation performance. However, the current study was designed to identify if there may be a relationship between colostrum intake and reproductive or lactation performance, rather than to identify the mechanism(s) behind such a relationship, but there are several possibilities.

One potential route through which adequate intake of colostrum could improve lactation performance is by improving the health and growth of heifers. Heifers that are healthy and have optimal pre- and post-weaning growth rates have been shown to have less incidence of dystocia and better lactation results than those with poor health and poor growth rates [39]. However, in the current study, there were only small differences in yearling weights between the colostrum intake categories, suggesting that it is unlikely that differences in growth rate were responsible for the differences in lactation results. 

Another potential explanation of the milk production differences is that the adequate uptake of bioactive components from colostrum may have an epigenetic effect on heifers. This may happen because bioactives in colostrum are (1) enhancing mammary gland development [13], (2) enhancing gut development and, therefore, feed conversion efficiency [40,41], and/or (3) amplifying metabolic and endocrine pathways to enhance lactation [13,42].

### 4.5. Study Limitations

Any results and conclusions from this project need to be viewed with consideration of the specific circumstances under which it was conducted. For example, approximately three quarters of the calves in this study came from a single, large commercial dairy, with the remainder coming from four smaller, family-owned dairies. In particular, this may have affected colostrum management, and the preservation of bioactives and maternal cells in the colostrum may not have equivalent across farms. In addition, as an observational, longitudinal study, there may be survivor bias. While these factors were addressed statistically, there is the possibility that because specific animals did or did not remain until the end of the project or that they come from a specific property, this may have influenced the final results and interpretations.

Obtaining data on the colostrum quality and total amount of colostrum fed to each calf also was not within the scope of this research project. Instead, the emphasis was on the result of colostrum intake, namely, the serum total protein concentration of each calf as measured in the first 1–7 days of life. It was an assumption made during this project that if a calf received adequate amounts of colostrum (as indicated by STP > 50 g/L), then it was also likely to have absorbed adequate amounts of other bioactive substances from colostrum. Serum total protein (STP) concentration was selected to estimate the transfer of passive immunity from colostrum because sample collection is minimally invasive for neonatal calves and results can be attained quickly and inexpensively. STP has been shown to accurately estimate the amount of IgG transferred to neonate calves from colostrum for up to 7 days after birth. Therefore, STP could be relied upon at least as an accurate reflection of their passive immunity status [28,29].

Other factors that may have influenced the results include the decision to exclude visibly sick or dehydrated calves from sampling, as dehydration concentrates the serum and can give a falsely high STP reading, and to sample calves born in different seasons and different years. However, the major limitation was that lactation data were only available from two of the five farms. Although this reduced the number of animals, the lactation data were of high quality and there were sufficient numbers of animals to allow statistical analysis. Nevertheless, further studies are needed in additional populations to confirm the milk yield findings herein, and if validated, then the specific bioactive substances in colostrum which may have epigenetic effects need to be identified and their physiological effects on developing heifers determined.

## 5. Conclusions

The results from this study indicate that neonatal colostrum intake does not have an important effect on the weights of heifers as yearlings, on their age at first and subsequent calvings or on the calving-to-calving interval. However, colostrum intake as a heifer neonate did affect the milk production performance as a cow during the first three lactations, suggesting potential epigenetic programming. Further study is needed though to explore this possible lactocrine programming effect in dairy heifers as a method to increase dairy cow productivity.

## 6. Glossary

Anti-Müllerian Hormone (AMH)—a reproductive hormone closely linked to the antral follicular count in female cattle.

Brix—a measurement of the total soluble solids in a liquid, as measured by refractometry and expressed as a percentage by weight.

Calf rearing—husbandry practices associated with raising a calf or calves.

Calving—the parturition process of cattle.

Cohort—a group of calves.

Colostrum—the first secretion from the mammary gland in cattle after calving. For the purposes of this study, it was defined as the mammary secretion collected in the first 12 h post-partum.

Cull/culling—removing cattle from the herd, due to death, illness, temperament, sale, herd changes or other factors.

Heifer—a young female bovine, before she has had a calf.

Inadequate (“poor”) transfer of passive immunity—when a young bovine does not intake enough colostrum to provide adequate immunity. It can be a result of poor-quality colostrum, insufficient volume of colostrum, colostrum being received too late after birth, etc.

Lactocrine hypothesis—the idea that a milk-based diet can affect the development of the neonate by means of bioactive substances, absorbed from the milk (or colostrum).

Muster—to gather cattle from the field or paddock into an area of confinement, usually to facilitate handling, sorting, treatments, etc.

Split calvings—where there is more than one calving season per property.

Transition milk—milk produced by the mammary gland of cattle 12 h to 4 days post-partum.

Yearling—cattle approximately one year of age. For the purpose of this study, yearling was defined as 9–16 months of age.

## Figures and Tables

**Table 1:** Number of heifers in adequate and poor neonatal colostrum intake categories (as estimated using serum total protein concentration (STP)) per farm with subsequent reproductive and lactation performance records.

	Farm 1		Farm 2		Farm 3		Farm 4		Farm 5		Total	
Neonatal STP category	Adequate	Poor	Adequate	Poor	Adequate	Poor	Adequate	Poor	Adequate	Poor	Adequate	Poor
Definition (g/L)	>50	≤50	>50	≤50	>50	≤50	>50	≤50	>50	≤50	>50	≤50
1–7 days of age (number and percentage)	1011 51%	968 49%	76 50%	76 50%	97 47%	108 53%	133 94%	9 6%	126 69%	57 31%	1443 54%	1218 46%
Total	1979		152		205		142		183		2661	
Yearling weight check (number and percentage)	776 54%	664 46%	32 56%	25 44%	72 50%	71 50%	131 94%	8 6%	80 76%	25 24%	1091 58%	793 42%
Total (number and percentage *)	1440 74%		57 38%		143 70%		139 98%		105 57%		1884 71%	
First calving (number and percentage)	537 56%	425 44%	26 53%	23 47%	59 50%	59 50%	128 94%	8 6%	83 67%	41 33%	833 60%	556 40%
Total (number and percentage *)	962 48%		49 32%		118 58%		136 96%		124 ^†^ 68%		1389 52%	

^†^ 19 heifers without weight data had first calving dates; * Percentage calculated based on the original total number of calves on each farm.

**Table 2:** Fat-and-protein-corrected milk (FPCM) means ± SE in kg per day in a standard 305-day lactation per lactation number for the serum total protein (STP) categories with *p*-values between poor and adequate colostrum intake.

	Farm 4		Farm 5		Both Farms Combined	
Neonatal STP category	Adequate	Poor	Adequate	Poor	Adequate	Poor
STP definition (g/L)	>50	≤50	>50	≤50	>50	≤50
First lactation FPCM	19.8 ± 0.1 ^a,b^	19.1 ± 0.1 ^a,b^	20.4 ± 0.1	20.1 ± 0.1	20.1 ± 0.1	19.6 ± 0.1 ^a,b^
Combined	19.4 ± 0.1 ^a,b^		20.2 ± 0.1		19.8 ± 0.0	
*p*-value	<0.001		0.014		<0.001	
Second lactation FPCM	27.0 ± 0.1 ^b,c^	25.9 ± 0.1 ^b,c^	29.8 ± 0.1	26.2 ± 0.1	28.4 ± 0.1	26.0 ± 0.1 ^a,c^
Combined	26.5 ± 0.1 ^b,c^		28.0 ± 0.1		27.2 ± 0.0	
*p*-value	<0.001		<0.001		<0.001	
Third lactation FPCM	28.6 ± 0.1 ^a,c^	27.7 ± 0.1 ^a,c^	28.0 ± 0.1	27.3 ± 0.1	28.2 ± 0.1	27.5 ± 0.1 ^a,c^
Combined	28.1 ± 0.1 ^a,c^		27.7 ± 0.1		27.9 ± 0.0	
*p*-value	<0.001		<0.001		<0.001	
Average FPCM	25.1 ± 0.1 *	24.2 ± 0.1 *	26.1 ± 0.1	25.5 ± 0.1	25.6 ± 0.0	24.4 ± 0.0
Combined	24.7 ± 0.0 *		25.8 ± 0.0		-	
*p*-value	<0.001		<0.001		<0.001	

^a,b,c^ Superscripts indicate differences at *p* < 0.001 between values within a single column (e.g., adequate category FPCM for first, second and third lactations); * An asterisk indicates a difference at *p* < 0.001 between the corresponding values of farms (e.g., adequate category FPCM combined between farm 4 and farm 5).

## Data Availability

Data available on request.

## Supplementary Materials

The following supporting information can be downloaded at: https://www.mdpi.com/article/10.3390/ani16213461/s1, Supplementary Table S1. Additional farm data, Supplementary Table S2. Neonatal serum total protein (STP) measurements and weight measurements at approximately one year of age, and Supplementary Table S3. Heifer/cow age at each recorded calving, per farm.

## Abbreviations

The following abbreviations are used in this manuscript:

CI	Confidence interval
FPCM	Fat-and-protein-corrected milk
HR	Hazard ratio
miRNA	MicroRNA
OR	Odds Ratio
STP	Serum total protein
