# Supplementary material for: Neonatal Colostrum Intake in Dairy Heifers Can Affect Lactation Performance
Source: Animals (Basel). 2026 Nov 2;16(21):3461. doi: 10.3390/ani16213461 (PMC13761776; doi:10.3390/ani16213461)
Supplement: Supplementary file 1 [file animals-16-03461-s001.zip › animals-4513476-supplementary.pdf]

Supplementary Table S1. Additional farm data

Farm	Cohort	Number of heifer calves in this study	Breed of calves	Calving season	Frequency of calf collection	Colostrum feeding	Date of "yearling" weight measurement	Max no of adult milkers
**1**	1	89	HF	Feb – Mar 2018	Twice a day	Unmeasured colostrum + transition milk	12/3/2019	2100
	2	636	HF	Jul – Oct 2018	Twice a day	Unmeasured colostrum + transition milk	3/9/2019	
	3	635	HF	Jul – Oct 2019	Twice a day	Unmeasured colostrum + transition milk	11/8/2020	
	4	619	HF	Jul – Oct 2020	Twice a day	Unmeasured colostrum + transition milk	7/9/2021	
**2**	5	152	HFx, Jx	Jul – Oct 2020	Once a day	Unmeasured colostrum	28/9/2021	410
**3**	6	205	HFx, Jx	Jul – Oct 2020	Once a day	Unmeasured colostrum + transition milk	17/9/2021	600
**4**	7	84	HF	Aug – Dec 2020	Twice a day	Colostrum BRIX > 22%	24/8/2021	420
	8	58	HF	Jan – May 2021	Twice a day	Colostrum BRIX > 22%	12/4/2022	
**5**	9	106	HF, HFx, J, Jx	Aug – Dec 2020	Twice a day	Unmeasured colostrum	15/2/2022 & 8/3/2022	500

10	77	HF, HFx, J, Jx	Jan – May 2021	Twice a day	Unmeasured colostrum	8/3/2022

HF = Holstein Freisian, HFx = Holstein Freisian crossbreed, J = Jersey, Jx = Jersey crossbreed.

Supplementary Table S2. Neonatal serum total protein (STP) measurements and weight measurements at approximately one year of age

	STP (mg/ml)			Weight at one year (kg)		
	n	Mean (median)	Minimum - maximum	n	Mean (median)	Minimum - maximum
**Farm 1**	1982	54.8 (52)	16 - 100	1440	261 (259)	147- 399
**Farm 2**	149	52.5 (54)	22 - 96	57	274 (280)	148 -365
**Farm 3**	205	51.8 (52)	32 - 86	143	274 (273)	176-358
**Farm 4**	142	62.8 (62)	42 - 92	139	289 (269)	190 - 391
**Farm 5**	183	56.7 (58)	39 - 92	105	268 (268)	152 - 392
**Total**	2661	55.5 (54.5)	16-100	1884	265 (262)	147 - 399

Supplementary Table S3. Heifer/cow age at each recorded calving, per farm

**Farm**	Age at 1st calving (days)			Age at 2nd calving (days)		
	n	mean (median)	minimum - maximum	n	mean	minimum - maximum
**1**	962	729 (725)	638 - 1443	794	1156	993-1965
**2**	49	720 (721)	685 - 792	48	1092	1048-1148
**3**	118	725 (724)	677 - 792	69	1108	1054 - 1457
**4**	136	751 (743)	631 - 938	108	1169	948 - 1464
**5**	124	841 (803)	705 - 1311	102	1227	1026 -1464
**Total**	1389	740 (728)	638 -1443	1121	1158	948 -1965

Supplementary Table S3 continued

Farm	Age at 3rd calving (days)			Age at 4th calving (days)			Age at 5th calving (days)		
	n	mean	minimum - maximum	n	mean	minimum - maximum	n	mean	minimum - maximum
**1**	581	1538	1324-2183	256	1913	1651-2372	65	2209	2099-2469
**2**	33	1453	1410-1499	na			na		
**3**	na			na			na		
**4**	25	1436	1286 - 1523	na			na		
**5**	27	1508	1398-1687	na			na		
**Total**	666	1529	1286 -2183	256	1913	1651 - 2372	65	2209	2099-2469
